# Rapid and Checkable Electrical Post-Treatment Method for Organic Photovoltaic Devices

**DOI:** 10.1038/srep22604

**Published:** 2016-03-02

**Authors:** Sangheon Park, Yu-Seong Seo, Won Suk Shin, Sang-Jin Moon, Jungseek Hwang

**Affiliations:** 1 Department of Physics, Sungkyunkwan University, Suwon, Gyeonggi-do, 440-746, Republic of Korea; 2 Division of Advanced Materials, Center for Solar Energy Materials, Korea Research Institute of Chemical Technology, Daejeon, 305-600, Republic of Korea

## Abstract

Post-treatment processes improve the performance of organic photovoltaic devices by changing the microscopic morphology and configuration of the vertical phase separation in the active layer. Thermal annealing and solvent vapor (or chemical) treatment processes have been extensively used to improve the performance of bulk-heterojunction (BHJ) organic photovoltaic (OPV) devices. In this work we introduce a new post-treatment process which we apply only electrical voltage to the BHJ-OPV devices. We used the commercially available P3HT [Poly(3-hexylthiophene)] and PC_61_BM (Phenyl-C_61_-Butyric acid Methyl ester) photovoltaic materials as donor and acceptor, respectively. We monitored the voltage and current applied to the device to check for when the post-treatment process had been completed. This electrical treatment process is simpler and faster than other post-treatment methods, and the performance of the electrically treated solar cell is comparable to that of a reference (thermally annealed) device. Our results indicate that the proposed treatment process can be used efficiently to fabricate high-performance BHJ-OPV devices.

In the 1980’s, semi-conducting organic polymers were discovered, which made it possible to fabricate new types of solar cells. Organic solar cells are flexible, low-cost devices that can be mass-produced, and their energy gap can be controlled via molecular engineering of the polymers. The energy gap is a very important parameter that affects the performance of the photovoltaic solar cells, and a thin active layer can absorb a large amount of light since the absorption coefficient of the organic molecules is generally quite large. However, the performance of organic photovoltaic cells has several limitations when compared to inorganic ones, including low efficiency, low stability, short lifetime, and low mechanical strength. For the past 30 years, intensive research has focused on organic photovoltaic (OPV) devices in order to improve the performance of organic solar cells.

P3HT [Poly(3-hexylthiophene)] and PC_61_BM (Phenyl-C_61_-Butyric acid Methyl ester) are the most commonly used donor and acceptor materials for OPV, respectively. P3HT is known to be a good absorber for photons with a wavelength shorter than 675 nm (a bandgap energy, *E*_*g*_ ~ 1.85 eV)^1^. Researchers have improved the power conversion efficiency of these materials using various techniques, including bulk-heterojunction fabrication^2^, annealing processes^3^,^4^,^5^,^6^, introduction of additives^7^,^8^, and so on. Annealing is one of the most common post-production treatment methods. In general, thermal annealing and solvent vapor (or chemical) treatment processes are used as post-treatment processes for OPV devices. Thermal annealing for a P3HT:PC_61_BM system was first introduced by F. Padinger *et al*.^3^. Thermally annealed polythiophene at a temperature higher than its glass transition temperature has been known to improve the crystallization, and this improved crystallinity enhances the hole conduction in polythiophene^9^. Solvent vapor annealing (SVA) treatment on a P3HT:PC_61_BM system was introduced by G. Li *et al*.^10^. In this method a spin-coated film contains the solvent for a given time, and the spin-coated wet film is then allowed to dry slowly in a covered glass petri dish. Recently, experimental studies [including atomic force microscopy (AFM)^11^,^12^, ultraviolet and X-ray photoemission spectroscopy (UPS, XPS)^13^,^14^, transmission electron microscopy (TEM)^12^,^15^, grazing-incidence wide-angle X-ray scattering (GIWAXS)^16^,^17^, and small angle neutron scattering^18^] showed that both thermal annealing and solvent vapor treatment processes improve the morphology and vertical phase separation of OPV, which consequently leads to a dramatic improvement in the performance of a P3HT:PC_61_BM bulk heterojunction system. Y. T. Wang *et al*. recently conducted a study with ultrafast time-resolved spectroscopy and found that a post-annealed device also had carriers with a longer lifetime in the acceptor HOMO level, which consequently produces a larger short circuit current density (*J*_*sc*_)^19^ than that obtained with untreated devices. A rapid annealing process was also introduced by using a high vapor pressure^20^.

In 2000, researchers used an electrical voltage to anneal polymer light emitting diodes (PLED)^21^. However, the authors applied a bias electrical voltage to a device that had already been treated using thermal annealing and improved the device efficiency from 1.43% (thermally annealed device) to 1.96% (with additional electrical voltage treatment). The authors explained that the electrical voltage that was applied aligned the polymer chains in the active layer to be parallel to the field, and this process improved the performance of the PLED. A couple of research groups applied similar methods to P3HT:PC_61_BM organic photovoltaic systems. F. Padinger *et al*. compared their thermally annealed device with another device that had been treated with thermal annealing under an external electrical voltage^3^. The latter device showed a slightly higher performance than the thermally-annealed one. B. P. Devi *et al*. applied a reverse (or backward) bias external electrical voltage to their device and showed around 9% relative improvement from 2.37% to 2.59% PCE with the reverse bias treatment^22^.

In this work we investigate the effect that electrical treatment with a forward bias electrical voltage has on the performance of P3HT:PC_61_BM system solar cells. We only applied an external electrical voltage to the solar cells for post-production treatment, and we were able to verify the completion of the electrical treatment process and achieved a slightly higher performance (or efficiency) than that with a thermally-annealed device with a much shorter processing time. The improvement in efficiency is of about 2.1 times when compared to the as-cast sample from our experiment. Our results show that the electrical treatment process is simple and rapid, can be verified, and achieves a high device performance. Therefore, this new process can be used for post-treatment of organic devices to improve their performance in a short period of time.

## Results

### Photovoltaic Properties of Solar Cell Devices

**Figure 1:**
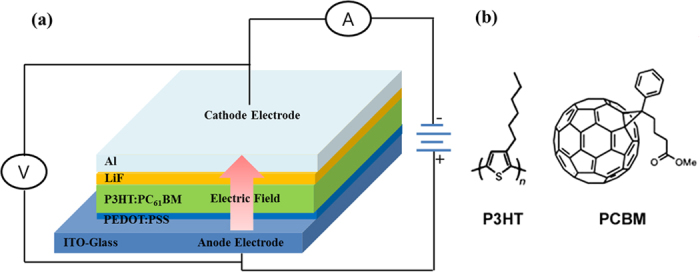
(**a**) Schematic drawing of the solar cell device with the wiring for the electrical post-treatment. (**b**) The molecular structures of P3HT and PC_61_BM.

**Figure 2:**
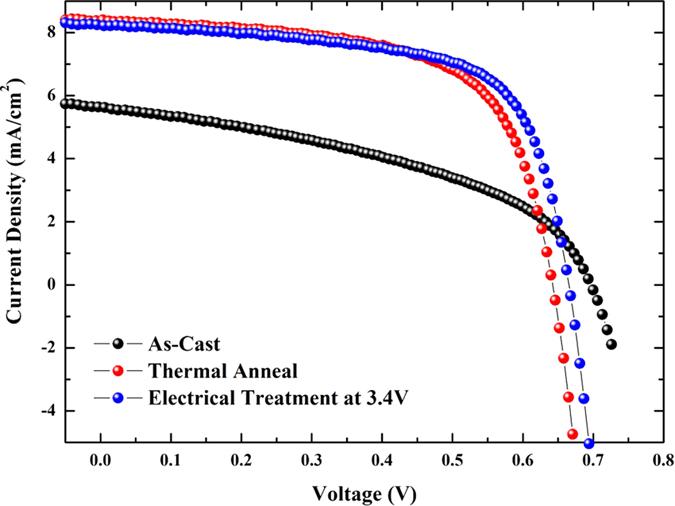
Current-voltage characteristics of our three samples. Black spheres are for the as-cast sample; red spheres are for the thermally annealed sample at 150 °C/30 min; blue spheres are for the electrically treated sample at +3.4 V/10 min.

We prepared the devices using three different post-treatment conditions (as-casted, thermally annealed, and electrically treated at +3.4 V) to compare the performance of each of the devices. The device architecture follows the conventional structure shown in Fig. 1a. In previous studies, the optimum annealing temperature and the thermal annealing time were 140 °C for 15 min^23^ and 150 °C for 30 min^24^ when using chlorobenzene (CB) as a solvent. For our thermal annealing process as a reference device, the optimum condition was 150 °C for 30 min with a CB solvent. Figure 2 shows the *J-V* characteristic curves of three different devices in air under an illumination of AM 1.5 G with 100 mW/cm^2^ using a solar simulator. The performance of the as-casted and the thermally annealed sample is consistent with previously reported results. The short circuit current density (*J*_*sc*_), the fill factor (*FF*), and the efficiency increases from 5.63 mA/cm^2^ (as-casted sample) to 8.39 mA/cm^2^ (thermally annealed sample), from 0.44 to 0.64, and from 1.72 to 3.44%, respectively. However, the corresponding open-circuit voltage (*V*_*oc*_) decreases from 0.70 to 0.65 V. All photovoltaic properties of three samples are provided in Table 1. A higher crystallization, better ordering of the intra-chain configuration, and an improvement in the vertical phase segregation of the thermally-treated sample improve the overall device efficiency^5^,^11^,^12^,^13^,^14^,^15^,^16^,^17^,^18^,^19^,^20^.

For the electrically treated device, the performance (or efficiency) depends on the voltage that is applied. To obtain the best performance, we prepared device samples by applying different voltages from +3 to +4 V with +0.2 V increment with the same treatment time (10 min) except +4 V case (5 min); for the +4 V case the device was damaged seriously when it was treated for longer time than 5 min. Further details of the experimental results are provided in Table S1 and Figure S1 in the Supplementary Information. The best performance was achieved when the applied voltage was +3.4 V as shown in Fig. 5a and Table S1. In this case, the power conversion efficiency (3.60%) is slightly higher than that (3.44%) for the thermally annealed device with device characteristics of 0.67 V (*V*_*oc*_), 8.23 mA/cm^2^ (*J*_*sc*_) and 0.66 (*FF*).

**Figure 3:**
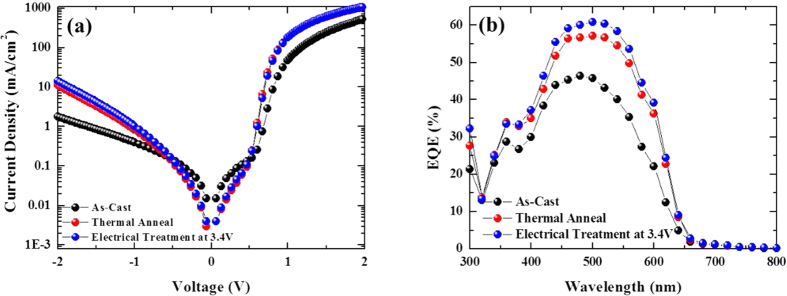
(**a**) Dark current densities and (**b**) IPCE spectra of our three samples. Black spheres are for the as-cast sample; red spheres are for the thermally annealed sample at 150 °C/30 min; blue spheres are for the electrically treated sample at +3.4 V/10 min.

In Fig. 3a, we display the measured dark current densities of our three devices (as-casted, thermally annealed, and electrically treated at +3.4 V). The series resistance (*R*_*s*_) and the shunt resistance (*R*_*sh*_) of device were estimated at 1 V and around 0 A, respectively. For the as-casted sample, *R*_*s*_ and *R*_*sh*_ are 21 Ωcm^2^ and 4,466 Ωcm^2^, respectively, and for the thermally annealed/electrically treated samples, *R*_*s*_ and *R*_*sh*_ are almost the same at 5.518 /5.638 Ωcm^2^ and 16,750 /14,888 Ωcm^2^, respectively. Therefore, there are no significant differences in the thermally annealed and electrically treated samples. The IPCE’s (or EQE’s) of the three devices are displayed in Fig. 3b. The IPCE’s of the thermally annealed and electrically treated devices dramatically increased over a wide spectral range when compared to that of the as-casted sample. The *J*_*sc*_ obtained from the measured IPCE for the as-casted, thermally-annealed, and electrically-treated samples were 5.44 mA/cm^2^, 7.20 mA/cm^2^, and 7.63 mA/cm^2^, respectively, and these values are consistent with the *J*_*sc*_ that was measured directly to accuracy within 10%. Data for our all devices are shown in Table S1 and Figure S2 in the Supplementary Information.

### Characteristics of Electrically Treated Devices

**Figure 4:**
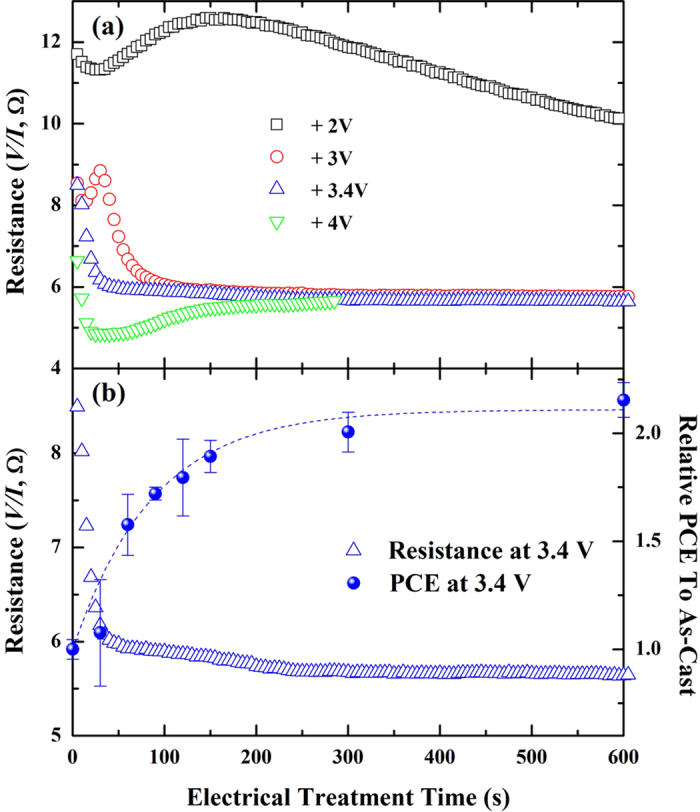
(**a**) Treatment time dependent device resistance of our four samples prepared by the electrical treatment process. Black open squares, red open circles, blue open triangles, and green open inverted triangles present electrically treated samples at +2, +3, +3.4, and +4 V, respectively. (**b**) The resistance and the relative power conversion efficiency of the electrically treated sample at +3.4 V as a function of the treatment time. The blue dash line is a guide to the eyes.

When an electrical voltage was applied to the device for electrical treatment, the applied voltage (*V*) and current (*I*) change over time. In Fig. 4a we show the measured resistance (*V/I,* Ω) as a function of the time measured from the beginning of the process for various applied electrical voltages (+2, +3, +3.4, and +4 V). For an applied voltage of +2 V, the resistance shows three different steps. The resistance initially decreases slightly for the first 30 s. Then, the resistance increases up to about 12.5 Ω at 150 s, and then decreases again with a moderate slope until the process is stopped. For an applied voltage of +3 V, the trend is similar to that for +2 V at up to 50 s but with shorter time scales; the resistance decreases down to 8 Ω at 15 s, then increases up to around 9 Ω at 30 s, and then decreases again and finally is saturated to a value of 5.7 Ω after around 100 s. The trend observed for +2 V and +3 V might be a result of the charge carrier dynamics in the active layer, including charge accumulation and depletion due to the applied electrical voltage. For an applied voltage of +4 V, the resistance shows a completely different behavior compared with +2 and +3 V cases; it decreases quickly down to its minimum value of 4.8 Ω at 20 s and then increases gradually up to a saturated value of 5.5 Ω. The resistance peaks that were observed for +2 V and +3 V were not observed for +4 V. At even higher applied voltages above +4 V, we observed an increase in the resistance immediately from the beginning of the process without any drops. As we mentioned previously we obtained the best efficiency (3.6%) when the cell had been treated with an applied voltage of +3.4 V. The resistance for +3.4 V shows a change in the slope at 10 s with 8 Ω (not very clearly shown in the figure scale), and it decreases more rapidly down to around 6 Ω at around 40 s and decreases slowly down to the saturated value of 5.7 Ω near 250 s without showing any upturns afterwards. In Fig. 4b we display the resistance and power conversion efficiency (PCE) of the device treated electrically with +3.4 V as a function of treatment time. As we have mentioned above, the resistance quickly decreases and becomes saturated near 250 s while the PCE increases and becomes saturated near 250 s where the resistance is saturated. This result indicates that the onset of the resistance saturation is a sign of the completion of our electrical treatment process.

**Figure 5:**
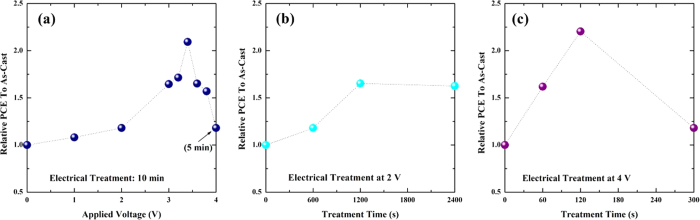
(**a**) Applied voltage-dependent PCE of samples prepared using the electrical treatment for 10 min except 4 V case (5 min). (**b,c**) Relative PCE enhancements of electrically treated cells with various treatment time at 2 V and 4 V, respectively.

We also investigated PCEs of devices treated at 2 V with longer treatment time than 600 s (or 10 min) and those at 4 V with shorter treatment time than 300 s (or 5 min) since time-dependent resistances for given applied voltages show non-trivial behaviors as shown in Fig. 4a. Further details of the experimental results for 2 V and 4 V cases are provided in Table S2 and Table S3 in the Supplementary Information. We display resulting treatment time-dependent PCEs of samples for 2 V and 4 V in Fig. 5b,c, respectively. For 2 V case the PCE seems to be peaked near 1200 s treatment time with about 1.6 times of the PCE of as-cast cell and then slowly decreases after 1200 s. We note that the maximum PCE for 2 V case is lower than that of the optimally treated cell at 3.4 V for 10 min. For 4 V the PCE seems to be peaked near120 s treatment time with around 2.1 times of that of as-cast cell and decreases rapidly after 120 s. We note that the maximum PCE for 4 V case is comparable to that of the optimally treated cell (3.4 V/10 min). After 120 s the electrode and sample surface become degraded very quickly and seriously while the resistance becomes slowly saturated as shown in Fig. 4a. Therefore, we expect that if we control another treatment parameter (the treatment time) appropriately with an applied voltage nearby 3.4 V we can fabricate high-performance cells with a similar PCE of the optimally treated cell (3.4 V/10 min).

The electrical voltage that is applied may heat up the device due to the Joule heating effect. To study this heating effect, we used a non-contact infrared temperature sensor to measure the temperature of our devices while they were electrically treated. The reliability of the measured temperature by the infrared sensor is discussed in detail in the Supplementary Information. We display maximum temperatures of the devices as a function of the applied voltage, along with the corresponding PCE in Figure S3b in the Supplementary Information. We note that the maximum temperature is the temperature when the electrical treatment is terminated. The maximum temperature increases almost linearly from room temperature up to 150 °C as the voltage increases from 2 V to 4 V (refer to Figure S3a). We note that the maximum temperature for 4 V case is taken with 5 min treatment time while the rest of maximum temperatures are taken with 10 min treatment time. The best efficiency was achieved at a maximum temperature of around 120 °C (a cell treated at 3.4 V for 10 min), which is lower than the temperature (150 °C) of our thermal annealing process. Our electrical treatment process seems to cause two effects: a modification in the morphology and vertical phase segregation by Joule heating and a modification in the alignment of the active layer due to the applied electrical voltage.

### Morphology Analysis of Electrically Treated Devices

**Figure 6:**
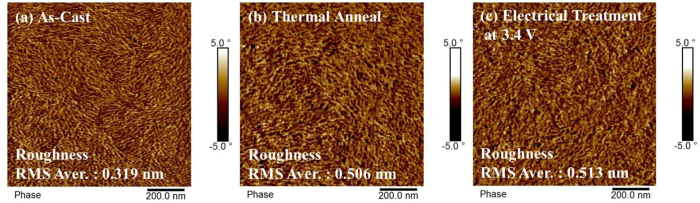
AFM phase images of three samples. (**a**) As-casted sample, (**b**) thermally annealed sample at 150 °C/30 min, and (**c**) electrically treated sample at +3.4 V/10 min.

We investigated the surface morphology of the three different devices by using an atomic force microscope (AFM) to capture surface images. We prepared two sets of three differently fabricated (as-cast, thermally annealed, and electrically treated) solar cells and took AFM images at three different positions from each cell. We note that our AFM images were taken from area nearby the electrode, particularly for the samples prepared using the electrical treatment process. In Fig. 6(a–c) we display representative AFM phase images of the as-casted, thermally annealed and electrically treated (at 3.4 V for 10 min) devices. We compared the phase image (1000 nm × 1000 nm area) of the as-casted sample (Fig. 6a) to that of the thermally-annealed one (Fig. 6b) and observed changes in the morphology of the P3HT/PC_61_BM surfaces of two samples; the P3HT fabric features become larger and the number of black dot PC_61_BM features increases in the thermally annealed sample. These results are consistent with results that were previously reported^9^,^14^. The average values of root-mean-square (RMS) roughness are shown in the figure. The RMS roughness (0.506 nm) of thermally treated cell increases 1.58 times (or 158%) compared to that (0.326 nm) of as-cast cell due to the modification of the surface morphology through thermal annealing^11^. P3HT polymers are known to be subjected to a deformation in the morphology along the vertical direction due to the mitigation of stress via thermal treatment. A previous study of the thermal annealing dynamics of polystyrene encaped with metal revealed this deformation mechanism^25^. The surface morphology of electrically treated cell (Fig. 6c) looks similar to that of the thermally annealed cell and its RMS roughness (0.513 nm) is slightly larger (1.4%) than that (0.506 nm) of the thermally annealed cell.

**Figure 7:**
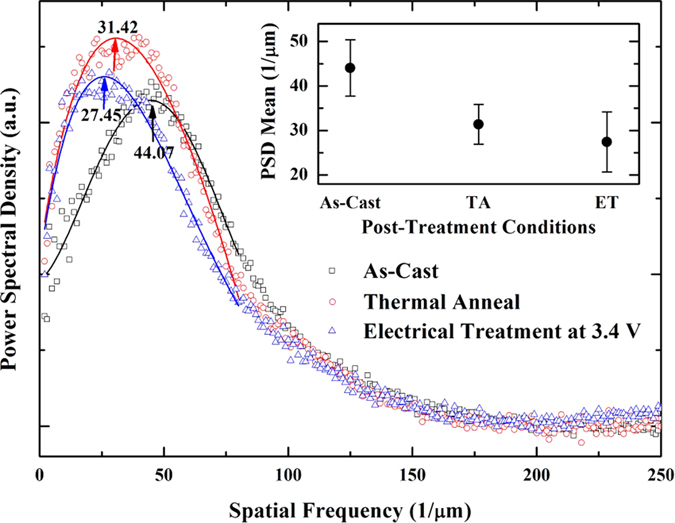
Power spectral densities of our three samples. Open symbols are experimental data from 2D-FFT images and solid lines are fitting curves. Black open squares are for as-cast sample; red open circles are for thermally annealed sample at 150 °C/30 min; blue open triangles are for electrically treated sample at +3.4 V/10 min. In the inset the average peak frequencies of our three samples in the average PSD data are displayed along with the statistical error bars. Here TA stands for thermal anneal at 150 °C/30 min and ET for electrical treatment at +3.4 V/10 min.

We investigate further the morphology of the sample surface by obtaining the power spectral density (PSD) from the AFM phase images. The average power spectral densities of our three differently prepared samples are displayed in Fig. 7. The characteristic length scales in the sample can be obtained from the PSD data; the peak in the PSD data can be used to obtain the major length scale, which is a reciprocal value of the spatial peak frequency. The peak positions in the average PSD data of the three differently prepared (as-casted, thermally annealed at 150 °C/30 min, and electrically treated at 3.4 V/10 min) samples are 44.07 μm^−1^, 31.42 μm^−1^, and 27.45 μm^−1^, respectively, and the corresponding major length scales are 22.6 nm, 31.8 nm, and 36.8 nm, respectively. The electrically treated sample shows the largest length scale among the three samples. We note that electrical (electric field) and thermal (Joule heating) effects are involved in the electrical treatment process. It is expected that the electrical treatment process produces a different molecular configuration from that of the sample treated using thermal annealing alone. The absolute length scales of as-casted and thermally annealed samples are larger than those obtained by Ma *et al*.^6^ from transmission electron microscopy (TEM) observations of the as-casted (14 nm) and thermally-annealed (20 nm) samples. The difference in the length scales obtained from the two techniques (AFM and TEM) might be attributed to their spatial resolution powers. However, the trend is similar; the thermally annealed sample shows a larger length scale than the as-casted one.

## Discussion

We have introduced a new post-treatment method that uses an external electrical voltage. We found a relationship between the applied voltage and the current, and these parameters (or the device resistance) can be monitored during electrical treatment and be used to determine when the treatment process has been completed. Therefore, we can finish the process at an appropriate time and protect the device from degrading due to the applied electrical voltage. Our electrical treatment process is simpler and faster than any other existing post-treatment processes and gives comparable device performance. We determined that the optimum applied voltage of the electrical treatment process with a given treatment time (10 min), which resulted in the highest efficiency, is +3.4 V. The device treated at this optimal voltage shows a slightly higher device performance than that of the thermally-treated device at 150 °C for 30 minutes. For +4 V case when we used shorter treatment time than 5 min we were able to achieve a comparable PCE to that of a cell treated under the optimal condition (+3.4 V/10 min) with a short period of time (2 min). Our analysis of the AFM images indicates that the geometrical properties (morphology) of the two differently prepared (thermally annealed and electrically treated) samples are not the same but quite similar: their RMS roughness and domain sizes (or characteristic length scales) are similar to each other. We expect that our electrical treatment method will be very useful for high-performance OPV device fabrication.

## Methods

### Device fabrication

Organic Photovoltaic (OPV) solar cells were fabricated as follows. The active layer was produced using a mixture of a donor [poly(3-hexythiophene), P3HT, purchased from Reike Materials Inc.] and an acceptor [phenyl[6,6]C_61_ butyric acid methyl ester, PC_61_BM, purchased from One Material Inc.]. ITO-coated glass plates were cleaned and used as substrates. The ITO glass was sonicated three times for 15 min in deionized water and detergent, cleaned with acetone, and rinsed again with isopropyl alcohol. An ultraviolet ozone treatment was conducted on the prepared ITO glass for 15 min to further clean the surface of the substrate. PEDOT:PSS (Clevios P) was filtered through a 0.45 *μ*m PTFE filter. Then, PEDOT:PSS was spin-coated on the ITO coated glass at 5500 rpm. The PEDOT:PSS films were dried at 140 °C for 15 min in air and were transferred into a nitrogen-filled glovebox. The P3HT:PC_61_BM (1:0.8 by weight) blend was dissolved in chlorobenzene (CB) and was stirred overnight. The blend was then spin-casted on top of the PEDOT:PSS film to obtain a ~100 nm-thick active layer. After drying for up to 2 hours, the devices were immediately loaded into a thermal evaporator where 0.5 nm-thick LiF and 100 nm-thick Al layers were consecutively deposited under a vacuum pressure of 2 × 10^−6^ torr. The device architecture and the molecular structures for P3HT and PC_61_BM are shown in Fig. 1a,b, respectively.

### Electrical treatment method

The solar cell device was electrically treated by applying an external electrical voltage from +1 to +5 V in the forward (or positive) bias direction. We note that the voltage in the backward direction does not produce a significant annealing effect^20^. As the external electrical voltage was applied, we monitored the surface temperature of the device using an infrared (IR) temperature sensor, and both the applied voltage and current were measured using digital multimeters (DMM, Agilent 34405a 5.5). The schematic circuit diagram of the experimental setup for electrical treatment is shown in Fig. 1a.

### Device characterization

We used a solar simulator with a Keithley 2400 source measurement to obtain the *J*-*V* characteristics of the device with a 3.2 mm × 3.2 mm mask (actual active area: 3 mm × 3 mm). We characterized our un-encapsulated solar cells in air under an illumination of AM 1.5 G, 100 mW/cm^2^ by using a solar simulator (McScience Inc.) equipped with a xenon arc lamp. We calibrated the intensity of the illumination by using a silicon diode certified by the National Renewable Energy Laboratory (NREL) and an integrated KG5. We measured the external quantum efficiency (EQE) using focused light output from a 100 W halogen lamp outfitted with a monochrometer and an optical chopper (McScience Inc.), and we used a reflective microscope objective for the focus.

## Additional Information

**How to cite this article**: Park, S. *et al*. Rapid and Checkable Electrical Post-Treatment Method for Organic Photovoltaic Devices. *Sci. Rep.* **6**, 22604; doi: 10.1038/srep22604 (2016).

## Supplementary Material

Supplementary Information

## Figures and Tables

**Table 1:** Photovoltaic properties of our three devices.

Devices		*V*_*oc*_(V)	*J*_*sc*_(mA/cm^2^)	*FF*	*PCE*(%)
Reference Devices	As-casted	0.70	5.63	0.44	1.72
	Thermally annealed (150 °C/30 min)	**0.64**	**8.39**	**0.64**	**3.44**
Electrically Treated Device	+3.4 (10 min)	**0.67**	**8.23**	**0.66**	**3.60**

All measurements were performed with a 3.2 × 3.2 mm^2^ mask.

## References

[b1] Dennler G., Scharber M. C. & Barbec C. J. Polymer-fullerene bulk-heterojunction solar cells. Adv.Mater. 21, 1323–1338 (2009).

[b2] Yu G., Gao J., Hummelen J. C., Wudl F. & Heeger A. J. Polymer photovoltaic cells: Enhanced efficiencies via a network of internal donor-acceptor heterojunctions. Science 270, 1789 (1995).

[b3] Padinger F., Rittberger R. S. & Sariciftci N. S. Effects of postproduction treatment on plastic solar cells. Adv. Funct. Mater. 13, 85–88 (2003).

[b4] Dittmer J. J., Marseglia E. A. & Friend R. H. Electron trapping in dye/polymer blend photovoltaic cells. Adv. Mater. 12, 1270 (2000).

[b5] Jiang X. . Specroscopy studies of photoexcitations in regioregular and regiorandom polythiophene films, Adv. Funct. Mater. 12, 587 (2002).

[b6] Ma W., Yang C. & Heeger A. J. Spatial fourier-transform analysis of the morphology of bulk heterojunction materials used in “Plastic” solar cells, Adv. Mater. 19, 1387–1390 (2007).

[b7] Peet J. . Method for increasing the photoconductive response in conjugated polymer/fullerene composites. Appl. Phys. Lett. 89, 252105 (2006).

[b8] Peet J. . Efficiency enhancement in low-bandgap polymer solar cells by processing with alkane dithiols. Nat. Mater. 6, 497 (2007). 17529968 10.1038/nmat1928

[b9] Zhao Y., Yuan G. X., Roche P. & Leclerc M. A calorimetric study of the phase transition in poly(3-hexylthiophene). Polymer 36, 2211 (1995).

[b10] Li G. . “Solvent Annealing” effect in polymer solar cells based on poly(3-hexylthiophene) and methanofullerenes. Adv. Funct. Mater. 17, 1636–1644 (2007).

[b11] Fang J. J., Tsai H. W., Ni I. C., Tzeng S. D. & Der Chen M. H. The formation of interfacial wrinkles at the metal contacts on organic thin films. Thin Solid Films 556, 294−299 (2014).

[b12] Park J. H., Kim J. S., Lee J. H., Lee W. H. & Cho K. Effect of annealing solvent solubility on the performance of poly(3-hexylthiophene)/methanofullerene solar cells. J. Phys. Chem. C 113, 17579−17584 (2009).

[b13] Tseng W. H. . Metal-induced molecular diffusion in [6,6]-phenyl-C61-butyric acid methyl ester poly(3-hexylthiophene) based bulk-heterojunction solar cells. Appl. Phys. Lett. 103, 183506 (2013).

[b14] Orimo A. . Surface segregation at the aluminum interface of poly (3-hexylthiophene)/fullerene solar cells. Appl. Phys. Lett. 96, 043305 (2010).

[b15] Ebadian S., Gholamkhass B., Shambayati S., Holdcroft S. & Servati P. Effects of annealing and degradation on regioregular polythiophene-based bulk heterojunction organic photovoltaic devices. Sol. Energy Mater. Sol. Cells 94, 2258−2264 (2010).

[b16] Treat N. D. . Interdiffusion of PCBM and P3HT reveals miscibility in a photovoltaically active blend. Adv. Energy Mater. 1, 82−89 (2011).

[b17] Kim H. J., Park J. H., Lee H. H., Lee D. R. & Kim J.-J. The effect of Al electrodes on the nanostructure of poly(3-hexylthiophene): Fullerene solar cell blends during thermal annealing. Org. Electron. 10, 1505−1510 (2009).

[b18] Yin W. & Dadmun M. A new model for the morphology of P3HT/PCBM organic photovoltaics from small-angle neutron scattering: Rivers and streams. ACS Nano 5 **(6)**, 4756–4768 (2011). 21563761 10.1021/nn200744q

[b19] Wang Y. T. . Use of ultrafast time-resolved spectroscopy to demonstrate the effect of annealing on the performance of P3HT:PCBM solar cell. ACS Appl. Mater. Interfaces 7, 4457–4462 (2015). 25692773 10.1021/am508091u

[b20] Jung B. Y., Kim K. M., Eom Y. M. & Kim W. C. High-pressure solvent vapor annealing with a benign solvent to rapidly enhance the performance of organic photovoltaics. ACS Appl. Mater. Interfaces 7, 13342−13349 (2015). 26061813 10.1021/acsami.5b01658

[b21] Lee T. W. & Park O. O. Effect of electrical annealing on the luminoous efficiency of thermally annealed polymer light-emitting diodes. Appl. Phys. Lett. 77, 3334 (2000).

[b22] Devi B. P., Thiyagu S. & Pei Z. “Electrical annealing” effect in bulk heterojunction polymer solar cells. Thin Solid Films 529, 54–57 (2013).

[b23] Kim Y. K. . Device annealing effect in organic solar cells with blends of regioregular poly(2-hexylthiophene) and soluble fullerene, Appl. Phys. Lett. 86, 063502 (2005).

[b24] Ma W., Yang C., Gong X., Lee K. H. & Heeger A. J. Thermally stable, efficient polymer solar cells with nanoscale control of the interpenetrationg network morphology, Adv. Funct. Mater. 15, 1617–1622 (2005).

[b25] Yoo P. J. & Lee H. H. Evolution of a stress-driven pattern in thin bilayer films: Spinodal wrinkling, Phys. Rev. Lett. 91, 154502 (2003). 14611470 10.1103/PhysRevLett.91.154502

