# Supplementary material for: Rapid and Checkable Electrical Post-Treatment Method for Organic Photovoltaic Devices
Source: Sci Rep. 2016 Mar 2;6:22604. doi: 10.1038/srep22604 (PMC4773849; doi:10.1038/srep22604)
Supplement: Supplementary Information [file srep22604-s1.pdf]

Rapid and Checkable Electrical Post-Treatment Method for Organic Photovoltaic Devices

Sangheon Park[1], Yu-Seong Seo[1], Won Suk Shin[2], Sang-Jin Moon[2]*, and Jungseek Hwang[1]*

[1]Department of Physics, Sungkyunkwan University, Suwon, Gyeonggi-do, 440-746, Republic of Korea
[2]Division of Advanced Materials, Center for Solar Energy Materials, Korea Research Institute of Chemical Technology, Daejeon, 305-600, Republic of Korea

Keywords: Organic Photovoltaic, Post-treatment, Thermal Annealing, Electrical Annealing, P3HT/PCBM

*Correspondence to [email: jungseek@skku.edu/ moonsj@krict.re.kr]

Table S1. *Photovoltaic properties of cells electrically treated in a fixed treatment time (10 min) with various applied voltages. As-cast and thermally treated cells are references. All measurements were performed with a 3.2×3.2 mm² mask. The values are our best results and values in the parentheses are averaged values of 4 solar cells.*

Devices		V_{oc} (V)	Measured J_{sc} (mA/cm²)	Calculated J_{sc} (mA/cm²)	FF	PCE(%)
Reference Devices	As-Cast	0.70 (0.70±0.004)	5.63 (5.38±0302)	5.44	0.44 (0.43±0.014)	1.72 (1.62±0.118)
	**Thermally Annealed (150 ℃/30min)**	**0.64 (0.66±0.000)**	**8.39 (8.19±0.181)**	**7.2**	**0.64 (0.64±0.001)**	**3.44 (3.36±0.075)**
Electrically Treated Devices	+ 1 V (10min)	0.69 (0.69±0.003)	5.83 (5.74±0.136)	5.97	0.46 (0.46±0.015)	1.86 (1.80±0.091)
	+ 2 V (10min)	0.66 (0.66±0.003)	6.09 (6.02±0.086)	5.73	0.50 (0.50±0.007)	2.03 (1.99±0.048)
	+ 3 V (10min)	0.64 (0.64±0.004)	7.14 (7.15±0.030)	6.76	0.62 (0.61±0.094)	2.82 (2.77±0.060)
	+ 3.2 V (10min)	0.65 (0.65±0.005)	7.17 (7.09±0.181)	-	0.63 (0.61±0.014)	2.95 (2.81±0.123)
	**+ 3.4 V (10min)**	**0.67 (0.66±0.003)**	**8.23 (8.14±0.081)**	**7.63**	**0.66 (0.65±0.007)**	**3.60 (3.52±0.067)**
	+ 3.6 V (10min)	0.68 (0.68±0.002)	6.79 (6.50±0.347)	-	0.61 (0.62±0.014)	2.84 (2.74±0.109)
	+ 3.8 V (10min)	0.69 (0.69±0.002)	6.58 (6.49±0.082)	-	0.60 (0.59±0.008)	2.70 (2.63±0.050)
	+ 4 V (5min)	0.69 (0.69±0.005)	6.00 (5.65±0.248)	5.4	0.49 (0.49±0.139)	2.03 (1.91±0.106)

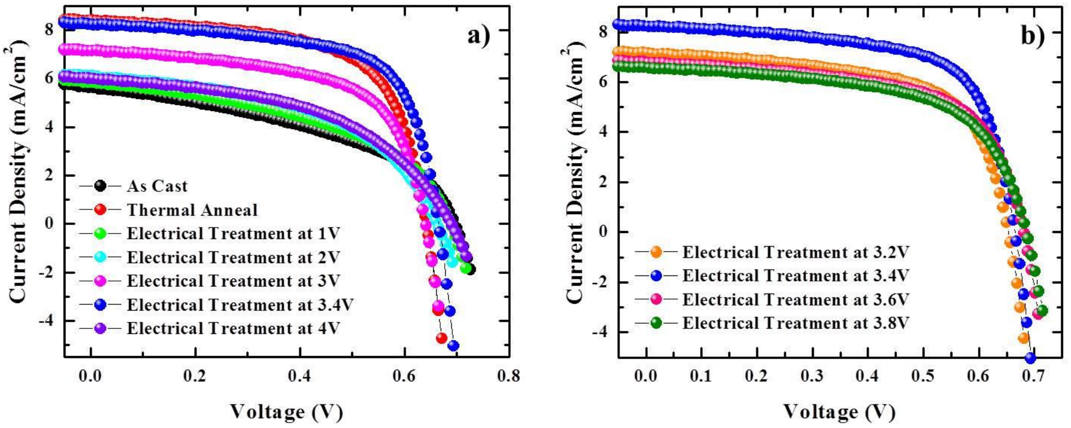

Figure S1. *J-V* characteristics of (a) all solar cell devices and (b) additional devices treated by the electrical post-treatment process with bias voltages from + 3.2 V to + 3.8 V with an increment + 0.2 V.

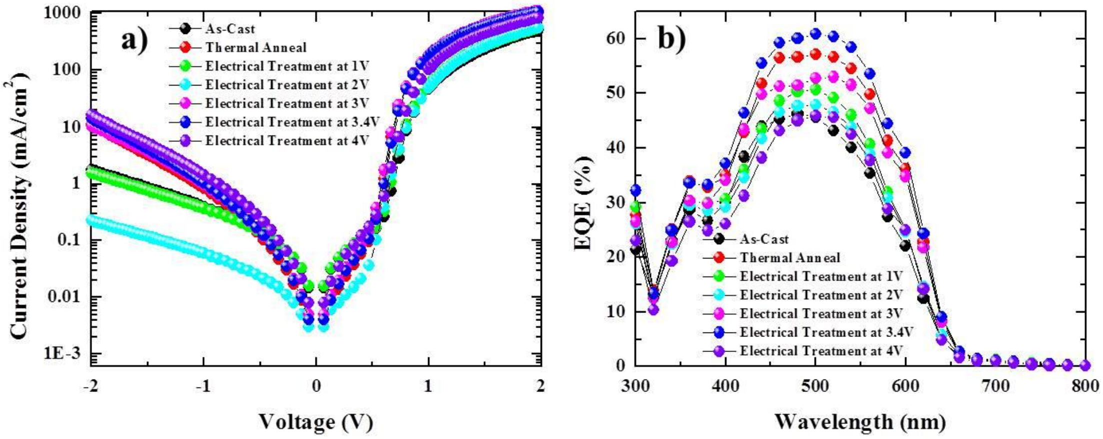

Figure S2. Dark current density data and IPCE spectra of all solar cell devices.

Table S2. *Photovoltaic properties of cells electrically treated at 2 V with various treatment time. As-cast and thermally treated cells are references. All measurements were performed with a 3.2×3.2 mm² mask. The values are our best results and values in the parentheses are averaged values of 4 solar cells.*

Devices		V_{oc} *(V)*	*Measured* J_{sc} *(mA/cm²)*	*FF*	*PCE(%)*
References Devices	*As-Cast*	*0.70*	*5.05*	*0.42*	*1.47*
		(0.70±0.001)	*(5.01±0.105)*	*(0.41±0.001)*	*(1.42±0.056)*
	Thermally Annealed *(150 ℃/30 min)*	***0.65***	***7.94***	***0.60***	***3.12***
		(0.65±0.007)	*(7.88±0.107)*	*(0.60±0.002)*	*(3.05±0.132)*
Electrically Treated Devices at 2 Voltage	*10 min*	*0.66*	*6.09*	*0.50*	*2.03*
		(0.66±0.003)	*(6.02±0.086)*	*(0.50±0.007)*	*(1.99±0.048)*
	20 min	***0.65***	***6.64***	***0.56***	***2.43***
		(0.65±0.001)	*(6.56±0.139)*	*(0.55±0.006)*	*(2.38±0.062)*
	40 min	*0.64*	*6.60*	*0.56*	*2.39*
		(0.64±0.002)	*(6.58±0.034)*	*(0.55±0.047)*	*(2.35±0.026)*

Table S3. *Photovoltaic properties of cells electrically treated at 4 V with various treatment time. As-cast and thermally treated cells are references. All measurements were performed with a 3.2×3.2 mm² mask. The values are our best results and values in the parentheses are averaged values of 4 solar cells.*

Devices		V_{oc} *(V)*	*Measured* J_{sc} *(mA/cm²)*	*FF*	*PCE(%)*
References Devices	*As-Cast*	*0.70*	*5.05*	*0.42*	*1.47*
		(0.70±0.001)	*(5.01±0.105)*	*(0.41±0.001)*	*(1.42±0.056)*
	Thermally Annealed *(150 ℃/30 min)*	***0.65***	***7.94***	***0.60***	***3.12***
		(0.65±0.007)	*(7.88±0.107)*	*(0.60±0.002)*	*(3.05±0.132)*
Electrically Treated Devices at 4 Voltage	*60 s*	*0.61*	*6.77*	*0.58*	*2.38*
		(0.61±0.002)	*(6.69±0.082)*	*(0.57±0.053)*	*(2.34±0.028)*
	120 s	***0.69***	***7.17***	***0.66***	***3.24***
		(0.69±0.002)	*(6.96±0.163)*	*(0.66±0.030)*	*(3.13±0.084)*
	300 s	*0.69*	*6.00*	*0.49*	*2.03*
		(0.69±0.005)	*(5.65±0.248)*	*(0.49±0.139)*	*(1.91±0.106)*

Reliability of infrared (IR) sensor

(1) Accuracy of temperature measured by the IR sensor

To check how reliable the temperature measured by the IR sensor is we performed a simple experiment as follows: we set a hot plate temperature to 100 ℃ in a glove box. We waited for around 10 min to get stabilization of the hot plate temperature and measured the stabilized temperature around the center of the hot plate 10 times by our IR sensor which is used for the experiment. The average temperature of 10 measurements is 98.98 ℃ and the statistical standard deviation (StDEV) is 4.32 ℃. The detailed results are shown in Table S4. We think that this accuracy may be good enough for our purpose.

Table S4. *The IR sensor reliability test*

Condition	*Measured Temperatures on the center of hot plate in glove box (℃)*										Average (℃)	*StDEV (℃)*
Value	104.5	108.1	99	100.1	96	99.1	95.6	96.9	95.7	94.8	98.98	4.32

(2) Input electrical energy during the electrical treatments

In order to estimate how much electrical energy is introduced into the device by the applied voltage we calculated the electrical energy (E_{elec}) for each given voltage using the following equation.

$$dE_{elec} = P(t)dt \quad \text{where } P(t) = V\, I(t)$$

$$E_{elec} = V \cdot \int_0^{t_D} I(t)\, dt$$

where $P(t)$ is the electrical power, V is the applied voltage, $I(t)$ is the current, and t_D is the treatment (or duration) time. In Figure S3a, we display calculated electrical energy as a function of applied voltage. For the calculations we took t_D as 300 s to make all treatment times be the same for different applied voltages; for the 4 V case we took dataset only up to 300 s, which is our shortest treatment time. If we consider dataset between 2 V and 4 V the electrical energy is almost linear to the voltage. However if we include data points below 2 V we can see the voltage-dependent energy deviates significantly from the liner behavior. Here

we note that the current is time-dependent and the electrical energy causes the Joule heating.

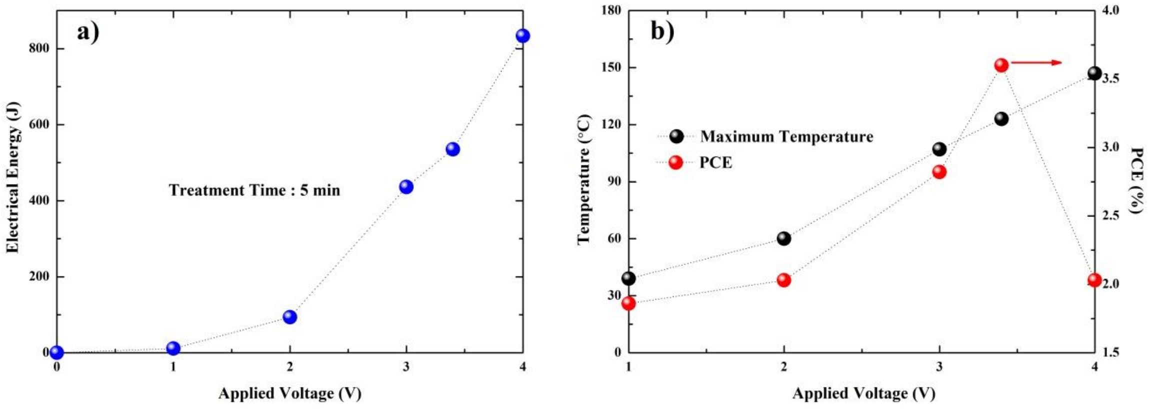

Figure S3. (a) Applied voltage-dependent electrical energy. (b) Applied voltage-dependent maximum temperature and PCE of the sample devices prepared using the electrical treatment with 10 min treatment time. We note that for 4 V case the treatment time is 5 min; 10 min treatment time may destroy the cell completely.

Furthermore we roughly estimated the device temperature increased by the applied voltage. The increased temperature (ΔT) can be written as

$$\Delta T = \frac{Q}{c\,m},$$

where Q is the total heat introduced by the applied voltage during the treatment, which is basically the same as the electrical energy, c is the (average) specific heat of the device, and m is the total mass of the device. We measured the total mass of the device; the average total mass of device is 1.7068 g (StDEV 0.0042 g with 6 samples). The device consists of several components: ITO glass, HTL, active layer, ETL and electrode. The (ITO) glass takes the most mass of the device with 1.6943 g (StDEV 0.0054 g). Therefore, we used the specific heat (~0.84 cal/g^{o}C) of a glass as that of the device for this estimation. The estimated temperature increase for the cell treated at 4 V/300 s is around 136 °C. Comparing with the measured temperature (150 °C as in Figure S3b) the estimated temperature (T_{room} + ΔT = 25 °C + 136 °C = 161 °C) agrees quite well with the measured one. Here T_{room} is the room temperature.
